# Work of the Foreign Relations Committee

**Published:** 1899-06

**Authors:** 


### Domestic Correspondence.

### WORK OF THE FOREIGN RELATIONS COMMITTEE.

Buffalo, N. Y., April 17, 1899.

To the Editor:

Sir,—You are well aware that as the direct result of the agitation in the National Association of Dental Faculties of the existence of fraudulent dental colleges, the sale of dental degrees by them at home and abroad, and the opportunities given for the outrageous traffic through the existence of laws permitting their legal incorporation, especially in the State of Illinois, an association of the universities and reputable literary and other institutions of learning was formed in that State for the purpose of securing a repeal of the obnoxious legislation. On the part of the Dental Faculty Association, the matter was in the hands of the Foreign Relations Committee, with power to take such action as it deemed best. The report made by that committee at the annual meeting of the Association held in Omaha, Neb., in August, 1898, had been extensively quoted, published, and circulated in the State of Illinois, and had been largely instrumental in bringing about this organization of the literary universities and colleges.

The Foreign Relations Committee very promptly secured competent legal counsel in the State of Illinois, and began a suit against the worst of the fraudulent dental colleges. In carrying on the suit the committee had the co-operation and assistance of the State Board of Health of Illinois, and the State Dental Examining Board. At the same time, in conjunction with the latter, it had prepared bills repealing the acts under which the fraudulent colleges had been incorporated.

The committee appointed by the Association of Literary Colleges had introduced more sweeping bills, and ours was to be used only in case of the failure of the more complete one of the universities.

The university bill aroused an opposition which proved fatal to it, whereupon the following was introduced, has been promptly passed, and is now part of the laws of the State of Illinois. Great credit is due the counsel of the Foreign Relations Committee, Mr. Walter Sayler, of Chicago, for his energetic and persistent action.

The suit of the committee against "The Independent Medical College," of Chicago, has been decided in our favor, but as was expected, it has been carried to a higher court. That concern was reported to own no less than eight different charters, and little could be expected until successful legislation had made them null and void. It was deemed best to carry on both attempts simultaneously, that the victory when it came might be the more complete.

W. C. Barrett,
*Chairman Committee.*

A BILL

For an act to amend Section 2 of "An Act concerning Corporations," approved April 18, 1872, in force July 1, 1872, as amended by act approved June 17, 1893, in force July 1, 1893.

SECTION 1. Be it enacted by the People of the State of Illinois represented in the General Assembly: That Section 2 of "An Act concerning Corporations," approved April 18, 1892, in force July 1, 1872, as amended by act approved June 17, 1893, be and the same is hereby amended so that the same shall read as follows:

Whenever any number of persons, not less than three nor more than seven, shall propose to form a corporation under this act, they shall make a statement to that effect, under their hands and duly acknowledged before some officer in the manner provided for the acknowledgment of deeds, setting forth the name of the proposed corporation, the object for which it is to be formed, its capital stock, the number of shares of which such stock shall consist, the location of its principal office, and the duration of the corporation, not exceeding, however, ninety-nine years, which statement shall be filed in the office of the Secretary of State. The Secretary of State shall thereupon issue to such persons a license as commissioners to open books for subscription to the capital stock of said corporation at such times and places as they may determine; but no license shall be issued to two companies having the same name:

Provided, that the Secretary of State is hereby empowered, and it shall be his duty, to revoke charters issued to corporations which authorize such corporations to confer degrees, diplomas, or other certificate or certificates of qualification in the science of medicine, pharmacy, or dentistry, upon the recommendation of the Attorney-General, such recommendation to be accompanied by affidavit that such corporation is conducting a fraudulent business or violating the terms of its charter; or the Attorney-General may in his discretion file a bill in chancery, in the name of the People of the State of Illinois, against any corporation conducting such fraudulent business or violating the terms of its charter, in any court having jurisdiction of the corporation and subject matter of such bill, and for an injunction to restrain said corporation from conducting its business fraudulently or violating the terms of its charter, and also for the dissolution of said corporation, and thereupon it shall be the duty of the court in which said bill is filed to hear and determine the same as in other cases in chancery.

And provided, further, that this act shall apply to schools, colleges, or universities which now or may hereafter be licensed in this State, notwithstanding any provisions that may exist in their charters.

